# New perspectives on the origins of the unique vocal tract of birds

**DOI:** 10.1371/journal.pbio.3000184

**Published:** 2019-03-28

**Authors:** Michael B. Habib

**Affiliations:** 1 University of Southern California, Los Angeles, California, United States of America; 2 Natural History Museum of Los Angeles County, Los Angeles, California, United States of America

## Abstract

Birds utilize a unique structure, called a syrinx, for the production of their vocalizations. The origins of the syrinx are not well understood. New work, utilizing first principles–based models, suggests that a key element in selection for the early syrinx might be the position of this vocal structure: although the larynx sits at the cranial end of the airway, the avian syrinx is located at the base of the airway at the split of the trachea to the lungs. This position may make the syrinx intrinsically more efficient, which might have been critical in the origin of this anatomical feature.

## Abstract 2

Birds use a unique structure called a syrinx for producing their calls, but its origins are not well understood. This Primer explores the implications of a new study suggesting that a key element in selection for the early syrinx might its position at the base of the airway, which may make it intrinsically more efficient.

## Primer

Vocalization is a complex behavior that involves a diverse array of anatomical structures. Most vertebrates utilize a larynx as the primary structure for production and control of voiced sounds. However, birds utilize a unique structure, called a syrinx, for the production of their vocalizations. The larynx in birds retains its function as a respiratory valve that protects the airway, but it lacks sound producing vocal folds. The syrinx is a complex structure that can have many more muscles than even the most complex larynx. The syrinx is a remarkable anatomical feature for multiple reasons. Unlike all other vertebrate vocal organs, the syrinx is not derived from a known valve precursor. The larynx appears to have been a fully functional sound production organ when it was replaced by the syrinx. As a result, there was presumably a prolonged evolutionary overlap of two functional sound producing organs associated with the origin of the syrinx. This type of prolonged functional overlap is rare for novel organs [1]. Finally, the syrinx is located in an unusual location for a vocal structure: although vocal organs in vertebrates are typically located at the cranial end of the airway, the syrinx is located at the base of the airway, where the trachea splits into two passages called main stem bronchii, one into each lung.

Syringeal anatomy gives many birds an exceptionally wide repertoire of sound production. Whereas vocalizations using a larynx are mostly modulated by control of a single pair of vocal folds, the syrinx utilizes a series of paired membranes that are variably tensioned by multiple muscle complexes, providing both passive and active oscillation control [2]. Because the syrinx sits at the bifurcation of the trachea, there are two merging passages for air through the base of the syrinx. Some birds are capable of producing different sounds from the left and right sides of the syrinx simultaneously [3]. In particular, in songbirds, the left and right syrinx can be operated and controlled separately; the sounds from each side can be produced simultaneously or independently. In some cases, each side is even specialized to different frequency ranges, expanding the total frequency range available. In essence, some songbirds can sing internal duets.

The origins of the syrinx are not well understood. Many features long considered unique to birds, particularly those related to feathered-wing flight, have been found to have existed in dinosaur groups outside the true avian lineage. The origins of avian flight were probably chaotic, with multiple near-aerial and/or fully aerial lineages of dinosaurs throughout the latter half of Mesozoic, with living birds representing the last living lineage of this experimentation [4]. The evolution of the syrinx may have followed a similarly complex path. If the syrinx followed an evolutionary path similar to the origins of flight, then the syrinx could have first evolved in nonavian dinosaurs (that is, in dinosaurs ancestral to birds but not themselves part of Aves). Conversely, the syrinx could be a feature that appear much later, in terms of both time and phylogeny. To look for which trajectory is more likely, one must turn to the fossil record, though preservation biases in fossils make tracing syringeal origins challenging. The early evolution of avian flight left a fossil record of musculoskeletal and feather anatomy, but airways and respiratory structures only very rarely preserve as fossils. As a result, direct evidence for the origins of the syrinx are lacking. Most reported syrinx fossils are geologically young (2.5 million years old or less). A single Eocene specimen is mentioned in the literature, but it was never described or illustrated. The oldest fossil of an avian syrinx discovered, to date, is from a specimen of *Vegavis* that dates to the latest Cretaceous (66 to 69 million years ago), described by Clarke and colleagues [5]. Although this is an exciting specimen that adds significantly to our understanding of syrinx evolution, it may not be representative of the origin of the syrinx. The syrinx in *Vegavis* is relatively derived, and the syrinx could have originated significantly earlier (in more basal birds). That said, based on *Vegavis*, it does now seem most likely that the syrinx originated within true birds (that is, rather than non-avian dinosaurs), well after the origin of feathered-wing flight [5].

Even if *Vegavis* is representative of early syringeal anatomy, the timing of the origin of the syrinx is only part of the story. The selection regime that drove the syrinx to functionally replace the larynx in birds has also been mysterious. Although the complex anatomy of the syrinx in many modern birds allows them to produce a particularly wide array of sounds, which seems intuitively advantageous, the earliest syrinx anatomy was probably less sophisticated. Something intrinsic to the syrinx must have therefore been involved in driving its takeover as the primary vocalization organ in birds.

New work by Riede and colleagues [6] suggests that the position of the syrinx may have played a key role in its origin and early evolution. They tackled the problem from a first principles perspective, looking at the core physical properties of acoustic systems in which sound modulation occurs at the end of a tube. Their models predict that placing the source of the sound at the base of the air passage is more efficient. A sound production organ converts aerodynamic energy to acoustic energy, and the way in which the sound production organ interacts with the primary air passage (the trachea, for land-living vertebrates) has a substantial effect on the efficiency of this conversion. A major contribution of Riede and colleagues is to demonstrate that sound-producing structures interact more favorably with the air passage when the sound production occurs at the base of the main airway rather than at the top of the airway. Or, from a flow perspective, it is favorable to have the sound source in an upstream position. The syrinx is in an upstream position, whereas the larynx is always in a downstream position, so the syrinx might have had an immediate advantage, even in a simple form, from its favorable interaction with the vocal tract (as compared to the larynx).

This efficiency boost depends on a number of factors, including the length of the vocal tract. In order to determine if position alone might give syrinx-based vocal production a boost to efficiency, Riede and colleagues [6] approached the problem from both a modeling and experimental perspective. Two modeling approaches were used to test whether positioning a sound source upstream or downstream of an elongated tube (the trachea) could have a significant effect on the efficiency of sound production. One challenge of testing this particular body of theory is that the modeling approaches cannot be replicated, in the strict sense, in vivo, because a syrinx or larynx cannot be moved freely along the tracheal tube (they are both fixed in their respective positions). Instead, Riede and colleagues complemented their models by manipulating the length of the airway and measuring the effects on the efficiency of the syringeal sound source. This allowed the authors to test predictions about the resonance between the trachea and syrinx.

Both the models and experimental work indicate that the resonance advantages of an upstream sound production organ are particularly significant when the airway is long. Even early birds had relatively long necks compared to typical vertebrates. As a result, they likely possessed a long airway, as living birds do. The longer trachea of birds compared to other vertebrates may have predisposed birds (or possibly near-bird dinosaurs) to the evolution of an upstream vocal organ [6]. This provides the first mechanically informed hypothesis for the origin of the syrinx.

The emphasis of this new investigation is on the modeling and theoretical work. This provides a framework of testable predictions that can be addressed with more extensive experiments. Although Riede and colleagues did utilize experiments with cadaveric birds, the samples and scope were necessarily limited. Future work will be able to build on their predictive models with more extensive and sophisticated experimentation. With an increasing array of in vivo imaging available, experimentation with live subjects may be able to elucidate some of the complex relationships between airway anatomy, syrinx structure, and acoustic resonance. Vocalization in birds is exceptionally complex. Understanding the evolution of syrinx diversity will require the inclusion of more variables. For now, however, Riede and colleagues have provided key evidence that the vocal organ position, and its interaction with airway length, represent critical variables in the evolution of vocalization.

This kind of theoretical work has implications beyond the discipline of vocal evolution. Biologists increasingly rely on a complex interplay between theory and experiment, in much the same way that physicists have done for decades. Biology is becoming more computational in nature, often merging classic biological observation with approaches classically used in engineering and applied physics. First principles and quantitative theory will likely become increasingly important, especially in an era when experimental work often produces immense data sets. Making the most efficient use of these data will require robust theoretical frameworks.
